# Bedside Ultrasonography versus Brain Natriuretic Peptide in Detecting Cardiogenic Causes of Acute Dyspnea 

**Published:** 2016

**Authors:** Keihan Golshani, Mehrdad Esmailian, Aniseh Valikhany, Majid Zamani

**Affiliations:** Emergency Department, Al-Zahra Hospital, Isfahan University of Medical Sciences, Isfahan, Iran.

**Keywords:** Ultrasonography, natriuretic peptide, brain, dyspnea, echocardiography, emergency service, hospital

## Abstract

**Introduction:**

Acute dyspnea is a common cause of hospitalization in emergency departments (ED).Distinguishing the cardiac causes of acute dyspnea from pulmonary ones is a major challenge for responsible physicians in EDs. This study compares the characteristics of bedside ultrasonography with serum level of blood natriuretic peptide (BNP) in this regard.

**Methods:**

This diagnostic accuracy study compares bedside ultrasonography with serum BNP levels in differentiating cardiogenic causes of acute respiratory distress. Echocardiography was considered as the reference test. A checklist including demographic data (age and sex), vital signs, medical history, underlying diseases, serum level of BNP, as well as findings of chest radiography, chest ultrasonography, and echocardiography was filled for all patients with acute onset of dyspnea. Screening characteristics of the two studied methods were calculated and compared using SPSS software, version 20.

**Results:**

48 patients with acute respiratory distress were evaluated (50% female). The mean age of participants was 66.94 ± 16.33 (28-94) years. Based on the results of echocardiography and final diagnosis, the cause of dyspnea was cardiogenic in 20 (41.6%) cases. Bedside ultrasonography revealed the cardiogenic cause of acute dyspnea in 18 cases (0 false positive) and BNP in 44 cases (24 false positives). The area under the ROC curve for bedside ultrasonography and BNP for differentiating the cardiogenic cause of dyspnea were 86.4 (95% CI: 74.6-98.3) and 66.3 (95% CI: 49.8-89.2), respectively (p = 0.0021).

**Conclusion:**

It seems that bedside ultrasonography could be considered as a helpful and accurate method in differentiating cardiogenic causes of acute dyspnea in emergency settings. Nevertheless, more study is needed to make a runaway algorithm to evaluate patients with respiratory distress using bedside ultrasonography, which leads to rapid therapeutic decisions in a short time.

## Introduction:

A cute dyspnea is a common cause of hospitalization in emergency departments (ED) (1). There are some clinical and para clinical measures such as patients’ history, physical examination and bedside ultrasonography that help physicians differentiate cardiac causes of acute respiratory distress from pulmonary ones. Some have introduced diagnostic rules such as the Framingham heart failure criteria. But, due to their low sensitivity and high specificity, they cannot distinguish acute heart failure from non-cardiac causes well (2). On the other hand, the sensitivity of electrocardiography (ECG) and bedside ultrasonography are not high enough to exclude the presence of cardiogenic pulmonary edema, as well (2, 3). Conventional echocardiography is an important diagnostic tool for screening and diagnosing heart failure but it is not available 24 hours a day and 7 days a week, additionally it needs many training time for skillful application. Brain natriuretic peptide (BNP) or N-terminal of the pro-hormone brain natriuretic peptide (NT-pro-BNP) has been shown to be able to rule out or establish the diagnosis of cardiogenic causes of acute respiratory distress (1). But, some diseases such as renal failure, critical illness, pulmonary heart disease, arrhythmia, anemia, valvular heart diseases and muscular diseases can affect the results of BNP measures (1, 4). Nowadays, bedside ultrasonography is integrated into the daily practice of emergency physicians, which helps them ameliorate the accuracy of their diagnoses and managements. Pulmonary ultrasonography helps us find lung pathologies in a short period of time, by interpreting the artifacts such as comet tails. These artifacts have high sensitivity, but low specificity in diagnosing pulmonary edema (5-7). In this study, we compare bedside ultrasonography with BNP in differentiating cardiogenic causes of acute dyspnea.

## Methods:

***Study Design***

This diagnostic accuracy study compares bedside ultrasonography with serum BNP levels in differentiating cardiogenic cause of acute respiratory distress from pulmonary sources. Echocardiography was considered as the reference test. The study was conducted in the emergency department of Al-Zahra Hospital, Isfahan, Iran, with more than 80000 patients admitted to the ED annually and a 40% admission rate. Before the initiation of the study, a 10-day local workshop was taught by a board-certified attending emergency medicine physician and a cardiologist, focusing on bedside ultrasonography with both didactic and hands-on trainings. At the time of admission, we obtained a written informed consent from participants or their legal guardians. The research was approved by institutional review board of ethic committee. 

***Participants***

The study population consisted of those who were admitted to the ED with acute onset of dyspnea between November 2013 and April 2014. Intubated and traumatic patients as well as patients with myocardial infraction and history of end stage renal disease were excluded. In addition, cases with the serum creatinine level above the normal range during laboratory evaluation were excluded. A checklist including demographic data (age and sex), vital signs, medical history, underlying diseases, serum level of BNP, as well as findings of chest radiography, chest ultrasonography, and echocardiography was filled for all participants.

***Procedure***

Patients were enrolled with convenience sampling method. On admission, after history taking and physical examination, an echocardiogram and a bedside ultrasonography were performed, and a blood sample was drawn for laboratory examinations (including BNP levels) during the first 15 minutes of admission. For measuring BNP level (by Bayer Diagnostic ADVIA centaur BNP assay method) 2 ml of peripheral venous blood sample was sent to the laboratory in a vacuum tube containing EDTA. Bedside ultrasonography was done with an (MyLab 25 Gold 2009) ultrasound machine (Biosound Esaote, Inc., Indianapolis, IN) using a CA430 Curved Array (3.5-5 MHz) and a LA523 Linear Array transducer (5.0 to 13.0 MHz) by trained emergency medicine residents within 30 minutes of admission. The majority of patients were in sitting or supine position in accordance with their comfort position. At first, ejection fraction (EF) of heart was estimated visually, via four-chamber, two-chamber, long axis and short axis views of the heart. EF was classified as good in case of EF ≥ 40% and low when EF was less than 40%. For performing ultrasonography, each hemi thorax was divided into 2 anterior (upper and lower) and 2 lateral zones. Anterior zone was the area between sternum and anterior axillary line while lateral zone was between anterior and posterior axillary lines. These zones were divided into upper and lower zones by the third intercostal space as a border. Lungs were scanned for presence of pleural sliding, B-Lines and A-Lines. Pleural sliding is the back and forth movement of visceral and parietal pleura. B-Lines are vertical pleural-based discrete, laser-like, hyper-echoic reverberation artifacts. They move with respiration simultaneously and extend to the bottom of the screen without fading. A-Lines are horizontal hyper-echoic reverberation lung artifacts that are separated from each other by an equal distance. For differentiating diastolic heart failure from pulmonary causes of acute respiratory distress, inferior vena cava (IVC) collapsibility index was measured. IVC collapsibility was defined as follows: maximum axial diameter of IVC minus minimum axial diameter of IVC divided by maximum axial diameter of IVC (IVC_max_- IVC_min_/IVC_max_). IVC diameters were measured at 2-3 centimeters from the right atrium, during expiration (IVC_max_) and inspiration (IVC_min_) with a curvilinear probe located longitudinally on sub-xyphoid area. Diastolic dysfunction was considered as source of acute dyspnea if EF was lower than 40% or EF was more than 40% but IVC collapsibility index was lower than 50%. Ultrasonography performance took 6 minutes for each patient. All echocardiography were performed by an expert cardiologist, who was blinded to clinical situation of patients. Final diagnosis of patients was decided based on clinical, laboratory, imaging and response to treatment findings by an expert cardiologist together with an internist.

***Statistical analysis***

Total sample size of this study was estimated 48 cases (Zα = 95%, p= 50%, d =15%). Data analysis was performed using SPSS software, version 20. Quantitative data were reported as frequency and percentage, and qualitative ones as mean and standard deviation. Sensitivity, specificity, positive predictive value (PPV) and negative predictive value (NPV) of bedside ultrasonography compared to BNP in differentiating cardiogenic causes of acute dyspnea were calculated. In addition, area under the receiver operating characteristic (ROC) curve with 95% confidence interval (CI) was calculated for the two diagnostic tests. Chi-square, Fisher’s exact test, and t-test were used for comparing the discrete and continuous variables. P-value < 0.05 was considered statistically significant.

**Table 1:** Baseline characteristics of participants based on cause of dyspnea

**Characteristics**	**Cause of dyspnea **		**P-value**
	**Cardiogenic**	**Non-Cardiogenic**	
**Age (year)**	64.7±21.2	68.1±13.8	0.6
**Sex**			
Male	9 (37.5)	15 (62.5)	0.55
Female	11 (45.8)	13 (54.2)	
History of CHF*			
Yes	5 (35.7)	9 (64.3)	0.748
No	15 (45.5)	18 (54.5)	
**Coronary artery disease**			
Yes	14 (43.8)	18 (56.3)	0.763
No	6 (37.5)	10 (62.5)	
COPD*			
Yes	4 (44.4)	5 (55.6)	1.000
No	16 (41)	23 (59)	
**Asthma**			
Yes	1 (33.3)	2 (66.7)	1.000
No	19 (42.2)	26 (57.8)	
**Hypertension**			
Yes	6 (40)	9 (60)	1.000
No	14 (42.4)	19 (57.6)	
**Jugular venous distension**			
Yes	3 (30)	7 (70)	0.488
No	17 (44.7)	23 (55.3)	
**Brain natriuretic peptide (pg/ml)**	2819±4095	1433±3029	0.184
**Ejection fraction (%)**	48±12	40±13	0.04

## Results:

48 patients with acute respiratory distress were evaluated (50% female). The mean age of participants was 66.94 ± 16.33 (28-94) years. Table 1 compares baseline characteristics of the studied patients. Based on the results of echocardiography and final diagnosis, the cause of dyspnea was cardiogenic in 20 (41.6%) cases. 

Bedside ultrasonography revealed the cardiogenic cause of acute dyspnea in 18 cases (0 false positive) and BNP in 44 cases (24 false positives). Table 2 summarizes final diagnosis of patients based on imaging, laboratory, and clinical findings. 

**Figure 1:**
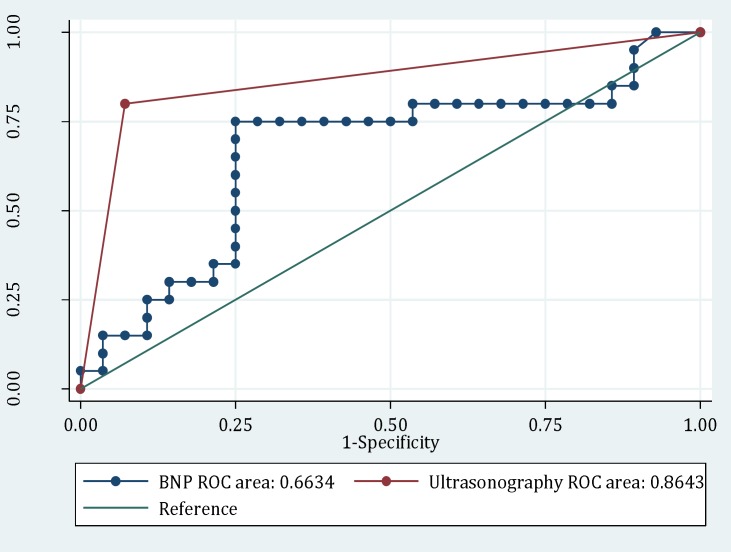
The area under the receiver-operating characteristic (ROC) curve for bedside ultrasonography and brain natriuretic peptide (BNP) for differentiating the cardiogenic causes of acute dyspnea (P = 0.0021

Final diagnosis of patients regarding the source of dyspnea was 100% compatible with echocardiographic results. The area under the ROC curve for bedside ultrasonography and BNP for differentiating the cardiogenic cause of dyspnea were 86.4 (95% CI: 74.6-98.3) and 66.3 (95% CI: 49.8-89.2), respectively (Figure 1, p = 0.0021). Table 3 summarizes the screening performance characteristics of the two studied diagnostic tests.

**Table 3:** Screening performance characteristics of bedside ultrasonography and brain natriuretic peptide (BNP

**Characteristics**	**Test (95% Confidence Interval)**		**P-value**
	**Ultrasonography**	**BNP** ^5^	
**Sensitivity**	80 (55.7-93.4)	70 (50.5-90.4)	0.0021
**Specificity**	92.8(75-98.7)	75 (54.7-88.5)	
**PPV** 1	88.9 (63.9-98)	68.1 (45.1-85.2)	
**NPV** 2	86.7 (68.3-95.6)	80.7 (60-92.6)	
**PLR** 3	8 (2.1-29.8)	2.1 (1.09-4.2)	
**NLR** 4	0.15(0.06-0.36)	0.23 (0.1-0.53)	
**Accuracy**	87.5 (74.75-95.27)	75 (60.4-86.36)	

1 . Positive predictive value;

2 . Negative predictive value;

3 . Positive likelihood ratio;

4 . Negative likelihood ratio.

5 . BNP’s characteristics were calculated in 703 pg/ml cut-offs.

**Table 2:** Final diagnosis of patients based on imaging, laboratory, clinical findings, and response to treatment

**Diagnosis**	**Number (%)**
**Asthma**	3 (3.6)
**Pulmonary edema**	19 (39.6)
**Pulmonary thromboembolism**	10 (20.8)
**Pneumonia**	4 (8.3)
**Myocardial infraction**	3 (6.3)
**COPD** *	5 (10.4)
**Pleural effusion**	1 (2.1)
**Emotional**	1 (2.1)

* COPD: Chronic obstructive pulmonary disease.

## Discussion:

Based on the results of the present study, bedside ultrasonography had better screening performance characteristics compared to BNP in detection of cardiogenic causes of dyspnea. The comparison of area under the ROC curve for the two tests showed significant accuracy of bedside ultrasonography in this regard. The causes of acute dyspnea divide into major categories of cardiac and non-cardiac. Distinguishing between these two categories is the first step in choosing therapeutic approach and still remains as one of the frequent challenges of emergency physicians. In this regard, some facilities such as BNP measurement and echocardiography can be helpful (8). Multiple studies have shown high diagnostic accuracy of serum BNP in ruling out heart failure as a cause of acute dyspnea (9-12). Yet, the high rate of cardiac and renal dysfunction in critically ill patients limits the discriminative role of BNP. In the present study, the sensitivity of BNP in 703 pg/ml cut-off was 70% while Arques et al. showed 86.4% and 68% sensitivity of BNP in 253 pg/ml and 480 pg/mL cut-offs, respectively (13). Serum BNP ≥ 22 pmol/L precisely reflected the final diagnosis of heart failure with 93% sensitivity and 90% specificity in Davis et al. study (14). In contrast to several studies in this regard, the accuracy of BNP in the present study was slightly low (15-18). This could be explained by the different cut-offs used for BNP in different studies. The accuracy of bedside ultrasonography in prediction of cardiac origin of dyspnea was known to be high (1). 

 Ultrasonography is more accurate in distinguishing free pleural effusion (19). In the majority of studies, bedside ultrasonography was performed by experienced operators within 10 minutes, and many studies claim completion of the exam within 3-5 minutes (20). In a study by Gargani et al. bedside ultrasonography in patients with acute dyspnea significantly correlated with their BNP values (1). Ultrasonography was shown to be accurate for differentiating cardiogenic acute dyspnea from those caused by primary pulmonary disease in the emergency setting (21). Multiple B-lines have been 100% sensitive and 92% specific in the diagnosis of pulmonary edema (22). In addition, the finding of diffuse multiple B-lines in bedside ultrasonography has had 95% specificity and 97% sensitivity in diagnosis of cardiogenic pulmonary edema (23). If these findings are confirmed in a large number of patients, bedside ultrasonography could become a valuable tool for situations where BNP is not available or in settings where other techniques are not readily available such as remote area, or even in out-of-hospital settings. If we use BNP along with bedside ultrasonography in order to optimize its diagnostic potential, we could reach the final diagnosis in less time. Yet, in addition to operator dependency, ultrasonography has some limitations such as application difficulties in obese patients, ribcage, and presence of subcutaneous emphysema (24).

## Conclusion:

It seems that bedside ultrasonography could be considered as a helpful and accurate method in differentiating cardiogenic causes of acute dyspnea in emergency settings. Nevertheless, more study is needed to make a runaway algorithm to evaluate patients with respiratory distress using bedside ultrasonography, which leads to rapid therapeutic decisions in a short time. 
